# Tigecycline in critically ill patients on continuous renal replacement therapy: a population pharmacokinetic study

**DOI:** 10.1186/s13054-018-2278-4

**Published:** 2018-12-17

**Authors:** A. Broeker, S. G. Wicha, C. Dorn, A. Kratzer, M. Schleibinger, F. Kees, A. Heininger, M. G. Kees, H. Häberle

**Affiliations:** 1 Department of Clinical Pharmacy, Institute of Pharmacy, University of Hamburg, Bundesstraße 45, 20146 Hamburg, Germany; 2 Institute of Pharmacy, University of Regensburg, Universitätstr. 31, 93053 Regensburg, Germany; 3 Hospital Pharmacy, University Hospital Regensburg, Franz-Josef-Strauß-Allee 11, 93053 Regensburg, Germany; 4 Department of Orthopaedics and Trauma, Hospital Ingolstadt, Krumenauerstraße 25, 85049 Ingolstadt, Germany; 5 Department of Pharmacology and Toxicology, University of Regensburg, Universitätsstr. 31, 93053 Regensburg, Germany; 6 Department of Infectious Diseases, Medical Microbiology and Hygiene, Division Hospital and Environmental Hygiene, Heidelberg University Hospital, Im Neuenheimer Feld 324, 69120 Heidelberg, Germany; 7 Department of Anesthesiology, University Hospital Regensburg, Franz-Josef-Strauß-Allee 11, 93053 Regensburg, Germany; 8 University Department of Anesthesiology and Intensive Care Medicine, University Hospital Tübingen, Hoppe-Seyler-Str. 3, 72076 Tübingen, Germany

**Keywords:** Tigecycline, Population pharmacokinetics, NONMEM, Dosing, Renal replacement therapy, CVVHD, CVVHDF

## Abstract

**Background:**

Tigecycline is a vital antibiotic treatment option for infections caused by multiresistant bacteria in the intensive care unit (ICU). Acute kidney injury (AKI) is a common complication in the ICU requiring continuous renal replacement therapy (CRRT), but pharmacokinetic data for tigecycline in patients receiving CRRT are lacking.

**Methods:**

Eleven patients mainly with intra-abdominal infections receiving either continuous veno-venous hemodialysis (CVVHD, *n* = 8) or hemodiafiltration (CVVHDF, *n* = 3) were enrolled, and plasma as well as effluent samples were collected according to a rich sampling schedule. Total and free tigecycline was determined by ultrafiltration and high-performance liquid chromatography (HPLC)-UV. Population pharmacokinetic modeling using NONMEM® 7.4 was used to determine the pharmacokinetic parameters as well as the clearance of CVVHD and CVVHDF. Pharmacokinetic/pharmacodynamic target attainment analyses were performed to explore the potential need for dose adjustments of tigecycline in CRRT.

**Results:**

A two-compartment population pharmacokinetic (PK) model was suitable to simultaneously describe the plasma PK and effluent measurements of tigecycline. Tigecycline dialysability was high, as indicated by the high mean saturation coefficients of 0.79 and 0.90 for CVVHD and CVVHDF, respectively, and in range of the concentration-dependent unbound fraction of tigecycline (45–94%). However, the contribution of CRRT to tigecycline clearance (CL) was only moderate (CL_CVVHD_: 1.69 L/h, CL_CVVHDF_: 2.71 L/h) in comparison with CL_body_ (physiological part of the total clearance) of 18.3 L/h. Bilirubin was identified as a covariate on CL_body_ in our collective, reducing the observed interindividual variability on CL_body_ from 58.6% to 43.6%. The probability of target attainment under CRRT for abdominal infections was ≥ 0.88 for minimal inhibitory concentration (MIC) values ≤ 0.5 mg/L and similar to patients without AKI.

**Conclusions:**

Despite high dialysability, dialysis clearance displayed only a minor contribution to tigecycline elimination, being in the range of renal elimination in patients without AKI. No dose adjustment of tigecycline seems necessary in CRRT.

**Trial registration:**

EudraCT, 2012–005617-39. Registered on 7 August 2013.

**Electronic supplementary material:**

The online version of this article (10.1186/s13054-018-2278-4) contains supplementary material, which is available to authorized users.

## Background

Tigecycline is the first example of a glycylcycline, a new derivative of tetracyclines, and an important option for the treatment of infections caused by multiresistant bacteria in the intensive care unit (ICU) [1, 2]. Acute kidney injury (AKI) is a frequent complication in ICU patients and may require renal replacement therapy (RRT). Continuous veno-venous hemodialysis (CVVHD) and continuous veno-venous hemodiafiltration (CVVHDF) are well established and very effective continuous RRT (CRRT) methods that can cause enhanced elimination of drugs, including antibiotics. Accordingly, higher doses of antibiotics may be necessary in patients with AKI during CRRT [3, 4]. No dosage adjustment of tigecycline is considered necessary in patients with renal impairment or in patients undergoing hemodialysis, but data on the pharmacokinetics (PK) during CRRT are lacking [5]. The aim of this study was to provide pharmacokinetic data in ICU patients undergoing CVVHD or CVVHDF, and to explore the potential need for dose adjustments by a probability of target attainment analysis.

## Methods

### Setting and study population

The study was performed in an anesthesiological ICU (40 beds) of a tertiary care hospital. Patients were included when they required RRT for AKI and were treated with tigecycline (loading dose of 100 mg followed by 50 mg twice daily). Major exclusion criteria were age > 85 or < 18 years, severe liver insufficiency (Child-Pugh C), acute pancreatitis, concomitant anti-coagulation therapy, or patients with a history of allergy to tigecycline. Written informed consent was obtained from either the patient or the legal representative.

### Continuous renal replacement therapy

All the equipment and solutions for CRRT were obtained from Fresenius Medical Care, Bad Homburg, Germany, except for calcium solutions which were provided by Serag-Wiessner, Naila, Germany. Patients were treated with CVVHD or CVVHDF using the MultiFiltrate system equipped with an Ultraflux AV 1000 S polysulfone membrane. For CVVHD, Ci-Ca® Dialysate K2, sodium citrate 4% and 0.5 M CaCl_2_ solution were used. Blood flow and dialysate flow were adjusted to body weight (< 90 kg/> 90 kg; 100/120 mL/min and 2000/2500 mL/h, respectively) [6]. The pre-filter dose of sodium citrate was adjusted to obtain a target concentration of ionized calcium post-filter of 0.25–0.35 mmol/L with a median flow rate of 176 mL/h (< 3% of blood flow) [7]. For CVVHDF, the multiBic® fluid was used for both dialysis and post-filter fluid replacement (post-dilution). Ultrafiltration rate (Q_Fil_) was 1 L/h. Anticoagulation was achieved with unfractionated heparin, targeting a 1.5-times greater activated clotting time compared with baseline.

### Sampling and drug analysis

Arterial blood samples were collected using Lithium Heparin Monovettes (Sarstedt, Nümbrecht, Germany) on day 4 of treatment with tigecycline after at least 24 h on CRRT. Samples were collected immediately before the start of infusion (time 0), after 1 h (i.e., the end of infusion), and then at 1.25, 1.5, 1.75, 2, 4, 6, 8, and 12 h. At the same time points, effluent was collected into polypropylene tubes from the effluent port of the CRRT circuit.

The blood was centrifuged (10 min, 3800 g), and the plasma as well as the effluent were stored at −70 °C until analysis. Tigecycline was determined by a validated high-performance liquid chromatography (HPLC)-UV method [8]. The free concentrations of tigecycline were measured in plasma after 1, 2, and 12 h. The limit of quantification in plasma was 0.05 mg/L tigecycline, and the intra- and interassay imprecision was < 6%. The respective values in effluent were 0.025 mg/L and < 9%, respectively.

### Pharmacometric analysis

For population PK modeling, NONMEM™ 7.4 (ICON Development Solutions, Hanover, MD, USA, FOCEI method) was utilized and executed via PsN (V 4.5.16) [9]. Interindividual variability was implemented on the structural PK parameters as follows:

1 $$ {P}_{k,i}={\theta}_k\times {e}^{\eta_{k,i}} $$

where *P*_*k,i*_ represents the estimated *k*th PK parameter for the *i*th individual calculated from the population PK parameter *θ*_*k*_ of the typical patient whilst *η*_*k*, *i*_ represents the deviation from the typical PK parameter assuming log-normal distribution.

The residual variability in an individual patient at each time point (i.e., the difference between individual model predicted (*Y*_*PRED*, *i*, *j*_) and the observed tigecycline concentration (*Y*_*OBS*, *i*, *j*_) for the *i*th subject at the *j*th time point) was estimated by a combined proportional (*ε*_*p*, *i*, *j*_) and/or additive (*ε*_*a*, *i*, *j*_) residual variability model:

2 $$ {Y}_{OBS,i,j}={Y}_{PRED,i,j}\times \left(1+{\varepsilon}_{p,i,j}\right)+{\varepsilon}_{a,i,j} $$

One- and two-compartment PK models with first-order disposition and elimination processes were fitted to the plasma data to determine the compartmental structure and the interindividual error model structure using ADVAN1 and ADVAN3 routines of NONMEM™. Allometric scaling models using total body weight with fixed [10] and freely estimated scaling parameters were evaluated.

The plasma PK model was extended to estimate the dialysis clearance. First principle dialysis modeling [11, 12] was used for this purpose as cumulated effluent concentration and volume measurements were not available.

Dialysis clearance (*CL*_*Dial*_) of CVVHD was calculated as follows using the effluent concentrations:

3 $$ {CL}_{Dial, CVVHD}={Q}_{Dial}\times \frac{C_{eff}}{C_{Pla}} $$

where *Q*_*Dial*_ represents the dialysate flow rate, *C*_*eff*_ represents the concentration of tigecycline in the effluent, and *C*_*Pla*_ represents the pre-filter plasma concentration.

For the CVVHDF method, the ultrafiltrate flow rate (*Q*_*Fil*_) needed to be considered in addition to *Q*_*Dial*_:

4 $$ {CL}_{Dial, CVVHDF}=\left({Q}_{Dial}+{Q}_{Fil}\right)\times \frac{C_{eff}}{C_{Pla}} $$

Mean saturation coefficients (C_eff_/C_pla_) for CVVHD indicating diffusion and for CVVHDF indicating convection, respectively, were calculated for each patient in the population PK model. Due to the very low citrate flow in relation to blood flow, no predilution correction of the CRRT clearance [13] was performed.

Age, sex, serum creatinine, creatinine clearance (Cockcroft-Gault), and bilirubin were tested as covariates on the body clearance (i.e., remaining total clearance separated from the dialysis clearance). All population PK parameters were modeled simultaneously.

Model selection was based on the likelihood ratio test (alpha = 0.05, change in degree of freedom = 1, i.e., drop in objective function value (dOFV) > 3.84) for nested models, Akaike information criterion (AIC) for non-nested models, and graphical criteria (goodness of fit plots (GOF) of population and individual prediction vs. observed concentrations, residual analyses, visual predictive checks (VPCs), *n* = 1000).

A mass balance analysis over 24 h under steady state was performed with the final model to investigate the impact of CVVHD; the low number of CVVHDF patients did not allow further investigation.

### Probability of target attainment analysis

The final pharmacometric model was used for clinical trial simulations (*n* = 500 simulations from the original study design) to investigate the impact of CRRT in the present population and to compare the present patient population to patients without renal failure and to healthy volunteers. Therefore, simulations from published models for patients with complicated skin and skin structure infections (cSSSI)/intra-abdominal infections (cIAI) [14] and healthy volunteers, respectively [15], were performed. Steady-state 24-h area under the concentration-time curve (AUC_24h_) and probability of target attainment (PTA) were calculated for the AUC_24h_/minimal inhibitory concentration (MIC) target for intra-abdominal infections of 6.96 [16].

## Results

### Patients, infections and pathogens

A total of 11 patients were included in the study (Table 1). The patients were treated with tigecycline due to cIAI (*n* = 10) or infection caused by *Acinetobacter baumanii* (*n* = 1). Relevant co-conditions were: liver failure or cirrhosis (four patients), liver transplantation (two patients), extracorporeal membrane oxygenation (ECMO; one patient). Two patients died during the follow-up period of the study. A total of 109 blood samples and 108 effluent samples were collected and used for the pharmacometric analysis, excluding two 12-h blood samples with very high tigecycline concentrations indicating that blood was taken after the start of the following infusion.

**Table 1:** Demographic and clinical details of the 11 patients included in the study

No.	Sex	Age (years)	Height (cm)	Weight (kg)	APACHE II	Serum creatinine (mg/dL)	Bilirubin (mg/dL)	Albumin (g/dL)	Protein (g/dL)	Co-condition
1^a^	M	69	176	69	21	1.7	2.3	2.1	6.4	
2^a^	F	47	160	70	21	1.2	9.2	2.8	5.9	Liver failure
3^a^	M	81	172	68	15	1.0	2.2	2.1	6.3	
4^b^	M	52	180	80	45	1.5	24.0	3.1	5.4	Liver failure
5^b^	M	78	178	70	25	0.5	3.5	2.6	3.5	Liver failure
6^a^	M	73	172	86	29	2.4	1.8	3.0	5.7	
7^b^	M	56	164	104	31	1.2	11.1	3.0	5.1	Liver cirrhosis
8^a^	M	37	182	85	21	1.3	43.3	2.8	4.3	Liver transplantation
9^a^	M	60	180	80	35	0.8	1.8	2.8	4.3	Liver transplantation
10^a^	M	74	170	73	30	0.7	2.2	2.6	5.6	
11^a^	M	75	180	80	30	0.7	0.7	2.7	5.6	ECMO
Median		69	176	80	29	1.2	2.3	2.8	5.6	
Minimum		37	160	68	15	0.5	0.7	2.1	3.5	
Maximum		81	182	104	45	2.4	43.3	3.1	6.3	

*APACHE* Acute Physiology and Chronic Health Evaluation, *ECMO* extracorporeal membrane oxygenation, *F* female, *M* male

^a^Continuous veno-venous hemodialysis (CVVHD); ^b^ continuous veno-venous hemodiafiltration (CVVHDF)

### Unbound fraction of tigecycline in plasma

The free plasma concentrations of tigecycline were determined after 1, 2, and 12 h (high, medium, low concentration), as tigecycline is reported to exhibit an inverse concentration-dependent plasma protein binding [17]. The decrease of the unbound fraction with increasing concentration was moderate (mean intraindividual coefficient of variation 4.9%, available as Additional file 1: Figure S1). The median unbound fraction of tigecycline in the patients was 61% (range 45–94%).

### Pharmacometric analysis

A two-compartment model with first-order disposition processes described the plasma concentration-time profiles adequately and was superior to a one-compartment model (dOFV of −113.77). Various interindividual variability (IIV) models were assessed. The best model included IIV on clearance, central volume of distribution and intercompartmental clearance, guided by lowest objective function value as well as graphical improvement. Shrinkage of the individual parameters towards the population mean was moderate (≤ 26%). A combined residual variability model (proportional and additive) was not supported (additive error tended to zero), so a proportional residual variability model was chosen.

Bilirubin (normalized by the population median of bilirubin, 2.3 mg/dL) as a covariate on clearance significantly improved the fit (dOFV = -5.71, *p* = 0.017, interindividual variability on clearance reduced from 58.6% to 43.6%) and was included in the final model (lower bilirubin concentrations corresponded to higher clearances). Allometric scaling with a fixed exponent did not improve the model significantly and was not included. The typical body clearance was 18.3 L/h. Individual clearance values varied from 9.3 L/h (10th percentile) to 19.1 L/h (90th percentile) depending on the bilirubin concentration (24 mg/dL to 1.8 mg/dL). One patient receiving ECMO was investigated by a case deletion study that showed no significant influence of this patient on the model parameters.

**Fig. 1:**
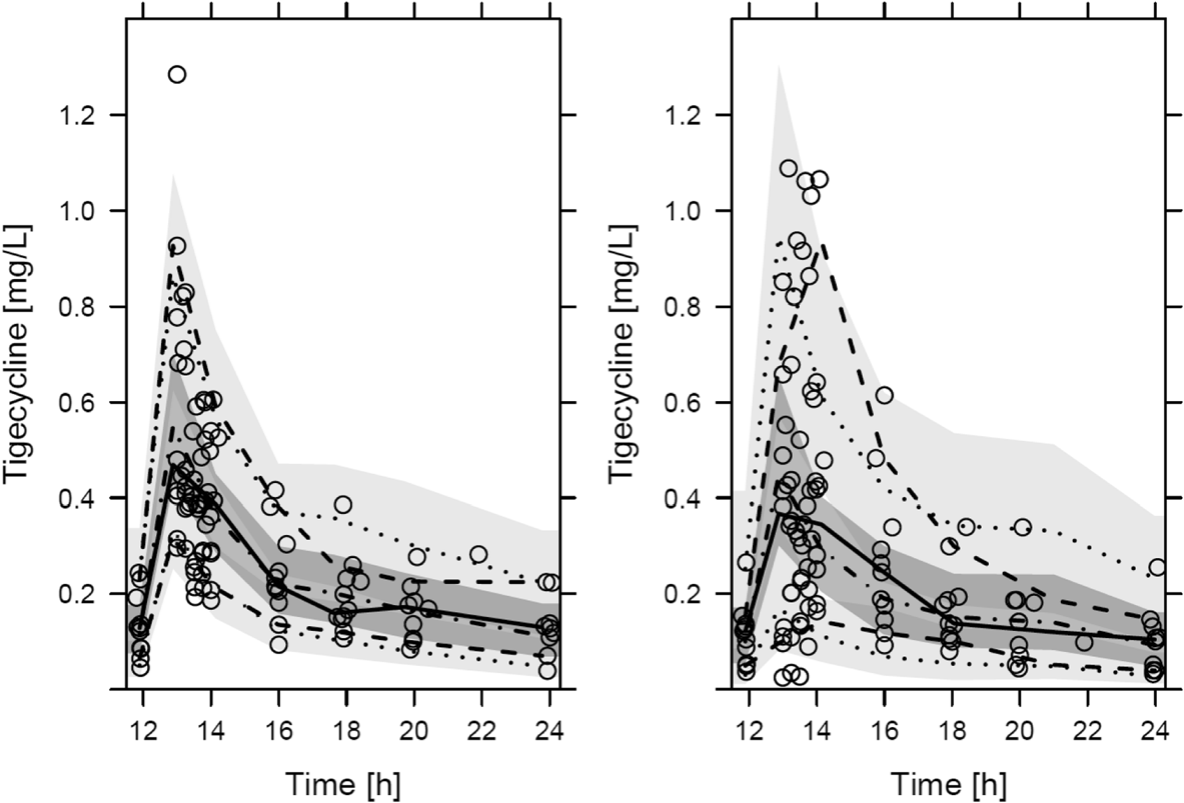
Visual predictive checks for the developed population PK model. Plasma concentrations (left) and effluent concentrations (right). Observed (solid lines) and predicted median (dashed-dotted lines) with 10th to 90th observed (dashed) and predicted (dotted) percentiles. Shaded areas: 95% confidence interval

The effluent measurements were added to the model and resulted in an estimated dialysis clearance of 1.69 L/h for CVVHD and 2.71 L/h for the CVVHDF method. The mean (±SD, interindividual variability) saturation coefficient was 0.79 ± 0.36 for CVVHD and 0.90 for CVVHDF (an IIV for this method was not supported by the data (IIV tended to zero during estimation)). The population pharmacokinetic parameters are presented in Table 2. The visual predictive check indicated high agreement between the observed and model-predicted tigecycline concentration-time profiles in plasma and effluent (Fig. 1).

**Table 2:** Typical pharmacokinetic parameters, unexplained interindividual variability and residual variability obtained from the pharmacometric analysis

Pharmacokinetic parameter	Estimate	RSE	95% CI^a^	Interindividual variability (%CV)
Clearance (L/h) = θ_1_ × ${\left(\mathrm{bilirubin}/2.3\right)}^{\theta_2}$				
θ_1_	18.3	11.0	13.2, 22.7	43.6
θ_2_	−0.29	33.1	−0.68, −0.10	
Central volume of distribution (V1) (L)	58.7	21.3	29.3, 101.6	110.9
Peripheral volume of distribution (V2) (L)	154	9.5	124.3, 196.8	–
Distribution clearance (Q) (L/h)	56.4	15.3	41.1, 76.6	41.8
Dialysis clearance CVVHD (L/h)	1.69	15.4	1.26, 2.27	43.5
Dialysis clearance CVVHDF (L/h)	2.71	8.9	2.31, 3.16	–
Residual variability				
σ_proportional, pre-filter plasma_ (%CV)	16.9	16.1	10.9, 21.7	–
σ_proportional, effluent_ (%CV)	40.6	13.1	30.4, 50.2	–

*CV* coefficient of variation, *CVVHD* continuous veno-venous hemodialysis, *CVVHDF* continuous veno-venous hemodiafiltration, *RSE* relative standard error (reported on standard deviation scale for variability parameters)

^a^95% confidence interval (CI) determined from a nonparametric bootstrap analysis (*n* = 1000)

The mass balance analysis revealed a median proportion of 11.2% (3.8% to 18.3%, 10th and 90th percentile) of tigecycline eliminated by hemodialysis within a dosing interval under steady state whereas the main proportion (88.8%) was eliminated by the body.

### Probability of target attainment

The simulated AUC_24h_ under steady state was (median, 10th to 90th percentile) 6.15 mg·h/L (3.39 mg·h/L, 11.13 mg·h/L) in the CVVHD patients in the present study. The simulated AUC_24h_ in cSSSI/cIAI patients was 5.65 mg·h/L (3.46 mg·h/L, 9.79 mg·h/L) and hence similar to the AUC_24h_ determined in our patient collective, but more variable than the AUC_24h_ of 6.24 mg·h/L (5.26 mg·h/L, 8.33 mg·h/L) in healthy volunteers.

**Fig. 2:**
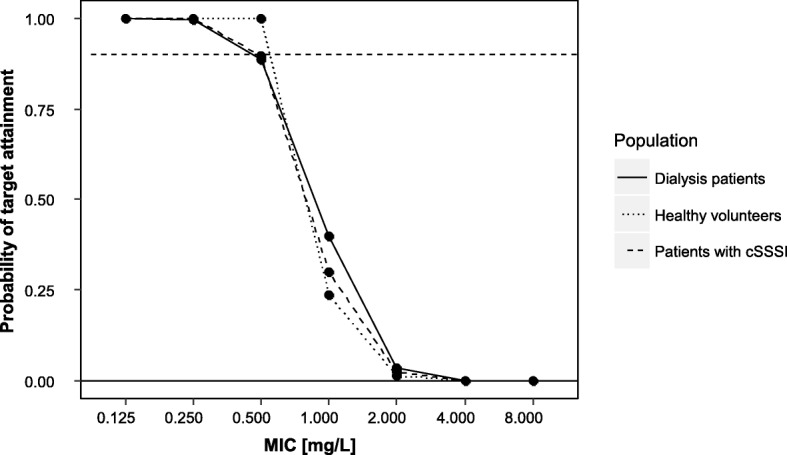
Probability of PK/PD target attainment analysis. Complicated intra-abdominal infections (AUC_24h_/MIC of 6.96) for dialysis patients of the present study compared with patients with cSSSI/cIAI (simulated from [15]) and healthy volunteers (simulated from [14]). Horizontal dashed line indicates PTA ≥ 0.9 considered as reliable target attainment. cSSSI complicated skin and skin structure infections, MIC minimal inhibitory concentration

Tigecycline standard dosing provided a high PTA of ≥ 0.9 for pathogens with MIC ≤ 0.5 mg/L to attain the PK/pharmacodynamic (PD) target for cIAI (AUC_24h_/MIC of 6.96) in our patient collective undergoing CRRT being comparable to cSSSI/cIAI patients (Fig. 2).

## Discussion

The present study investigated the steady-state pharmacokinetics of tigecycline 50 mg twice daily in ICU patients with AKI during CVVHD or CVVHDF. The pharmacometric analysis was performed using a population pharmacokinetic model employing effluent measurements for estimation of CRRT clearance. The clearance of the CVVHDF method was estimated to be more efficient (2.71 L/h) than the CVVHD method (1.69 L/h).

The typical PK parameters in our collective of CRRT patients were in close approximation to those determined in healthy volunteers [15] and cSSSI/cIAI patients [14]. This was also reflected in the clinical trial simulations, where the obtained mean AUC_24h_ under a steady state was similar between our CRRT patients, cSSSI/cIAI patients [14], and healthy volunteers [15]. Notably, the variability in AUC_24h_ (10th to 90th percentile) in healthy volunteers was markedly lower (5.26 mg·h/L, 8.33 mg·h/L) than in our patients (3.39 mg·h/L, 11.13 mg·h/L).

The covariate inclusion of bilirubin as a marker for hepatic function seemed reasonable and a previous pharmacokinetic analysis also identified bilirubin as a significant covariate, even though it was not included in their final model [14]. The use of ECMO in one patient had no significant influence on the model, which is in agreement with the case report of Veinstein et al. [18]. Additionally, the inclusion of the effluent into the pharmacometric model allowed us to distinguish between body (hepatic) clearance and dialysis clearance. The mass balance analysis using the pharmacometric model clarified that CRRT clearance is a minor factor for drug elimination (11.2%) and on a comparable level to renal elimination (13% according to the summary of product characteristics (SPC)).

According to the manufacturer’s SPC (SPC Tygacil 50 mg powder for solution for infusion, Pfizer Limited, Sandwich, UK), the in-vitro plasma protein binding of tigecycline ranges from 71% to 89% at concentrations observed in clinical studies (0.1 to 1.0 mg/L), corresponding to an unbound fraction (*f*u) of only 11–29%. These protein binding data have been suggested as an explanation for the poor elimination of tigecycline by intermittent hemodialysis [19]. However, the mean saturation coefficient of 0.79 for CVVHD or 0.90 for CVVHDF as found in the present study indicates good transfer of tigecycline through the dialysis membrane, and is a strong argument against a high plasma protein binding of tigecycline. Indeed, recent in-vitro investigations revealed that the protein binding of tigecycline in human plasma is rather moderate with a *f*u of about 50–70% at therapeutic concentrations [8]. Using this ultrafiltration method, which (in contrast to other methods) mimicked physiological conditions during ultrafiltration, the mean unbound fraction of tigecycline in the plasma of the study patients was determined to be 45–94%, in line with the saturation coefficient for CVVHD. In conclusion, the small extracorporeal clearance of tigecycline of only 11.2% of the administered dose is due to the high volume of distribution of tigecycline and not due to high plasma protein binding.

The PK/PD target attainment in our patients was similar to that in cSSSI/cIAI patients using the published model by Van Wart et al. [14]. Considering the reported PK/PD target for AUC_24h_/MIC of 6.96 (cIAI) [16], the standard dose of tigecycline (100 mg followed by 50 mg b.i.d.) would be considered appropriate for pathogens with a MIC value of up to 0.5 mg/L.

Some limitations of our study have to be acknowledged. The data have a pilot character, as they are based on eight patients receiving CVVHD and three patients receiving CVVHDF. However, it should be acknowledged that the studied collective represents a vulnerable population and pharmacokinetic data are lacking, and the chosen approach of a pharmacometric analysis maximized the information content drawn from the population. Still, it would be desirable to study longer time periods across several dosing occasions in future studies to detect potential time-dependencies in the pharmacokinetics. Protein binding of tigecycline is affected by divalent cations such as calcium [8]; hence, citrate anticoagulation within the extracorporeal circulation might theoretically affect its transfer through the dialysis membrane. Moreover, tigecycline can adsorb to plastic labware [8] and apparently also to dialysis membranes [19]. In one patient, we observed a time delay in the effluent concentrations which may have been caused by adsorption losses, e.g., after changing the filter, and which would have resulted in an underestimated dialysis clearance. Since the delay indicated a reversible or saturable binding, probable adsorption losses did not impact the estimated dialysis clearance significantly, and certainly did not influence systemic drug exposure. However, potential adsorption of tigecycline to other membrane types and tigecycline dialysis clearance in other RRT systems should be investigated in future studies. For the PK/PD target attainment analysis, our analysis focused on total rather than unbound AUC_24h_ due to a lack of reliable clinical breakpoints for *f*AUC_24h_/MIC. Future clinical studies considering unbound concentrations for PK/PD target attainment are highly warranted. The use of reliable techniques in these trials will be crucial to ensure that the determined *f*AUC_24h_/MIC will be not biased by the methodology utilized to determine the unbound fraction.

## Conclusions

The pharmacokinetic parameters of tigecycline are not significantly influenced by CRRT. The probability of target attainment was similar in the present patient collective receiving CRRT compared with patients with no AKI, indicating that no dose adjustment seems necessary in CRRT.

## Additional file

Additional file 1: **Figure S1.** Unbound fraction of tigecycline in the plasma of 11 patients undergoing CRRT. Solid line = CVVHD, dashed line = CVVHDF. (DOCX 59 kb)

## Abbreviations

AIC: Akaike information criterion
AKI: Acute kidney injury
AUC_24h_: Area under the concentration-time curve in 24 h
C_eff_: Effluent concentration of tigecycline
cIAI: Complicated intra-abdominal infections
CL: Clearance
CL_Dial_: Dialysis clearance
C_Pla_: Plasma concentration of tigecycline
CRRT: Continuous renal replacement therapy
cSSSI: Complicated skin and skin structure infections
CVVHD: Continuous veno-venous hemodialysis
CVVHDF: Continuous veno-venous hemodiafiltration
dOFV: Drop in objective function value
ECMO: Extracorporeal membrane oxygenation
*f*u: Unbound fraction
HPLC: High performance liquid chromatography
ICU: Intensive care unit
IIV: Interindividual variability
MIC: Minimal inhibitory concentration
NONMEM: Nonlinear mixed effects modeling
PK: Pharmacokinetics
PTA: Probability of target attainment
Q_Dial_: Dialysate flow rate
Q_Fil_: Ultrafiltration flow rate
RRT: Renal replacement therapy
SPC: Summary of product characteristics
VPC: Visual predictive check
