# Supplementary material for: Tigecycline in critically ill patients on continuous renal replacement therapy: a population pharmacokinetic study
Source: Crit Care. 2018 Dec 17;22:341. doi: 10.1186/s13054-018-2278-4 (PMC6296114; doi:10.1186/s13054-018-2278-4)
Supplement: Supplementary file 1 — Figure S1. Unbound fraction of tigecycline in the plasma of 11 patients undergoing CRRT. Solid line = CVVHD, dashed line = CVVHDF. (DOCX 59 kb) [file 13054_2018_2278_MOESM1_ESM.docx]

**Population pharmacokinetics of tigecycline in critically ill patients on continuous renal replacement therapy**

**Authors:**

A. Bröker^1^, S.G. Wicha^1^, C. Dorn^2^, A. Kratzer^3^, M. Schleibinger^4^, F. Kees^5^, A. Heininger^6^, M.G. Kees^7^, H. Häberle^8^

**Affiliations:**

^1^Department of Clinical Pharmacy, Institute of Pharmacy, University of Hamburg, Bundesstr. 45, 20146 Hamburg, Germany

^2^Institute of Pharmacy, University of Regensburg, Universitätstr. 31, 93053 Regensburg, Germany

^3^Hospital Pharmacy, University Hospital Regensburg, Franz-Josef-Strauß-Allee 11, 93053 Regensburg, Germany

^4^Department of Orthopaedics and Trauma, Hospital Ingolstadt, Krumenauerstraße 25, 85049 Ingolstadt, Germany

^5^Department of Pharmacology and Toxicology, University of Regensburg, Universitätsstr. 31, 93053 Regensburg, Germany

^6^Department of Infectious Diseases, Medical Microbiology and Hygiene, Division Hospital and Environmental Hygiene, Heidelberg University Hospital, Im Neuenheimer Feld 324, 69120, Heidelberg, Germany

^7^Department of Anesthesiology, University Hospital Regensburg, Franz-Josef-Strauß-Allee 11, 93053 Regensburg, Germany

^8^University Department of Anesthesiology and Intensive Care Medicine, University Hospital Tübingen, Hoppe-Seyler-Str. 3, 72076 Tübingen, Germany

**Supplementary Figures**

**Figure S1:** Unbound fraction of tigecycline in plasma of 11 patients undergoing CRRT. Solid line = CVVHD, dashed line = CVVHDF.
